# Adapting to Change: The Evolution of the Specialty Training Level 3 (ST3) Trauma and Orthopaedics Selection Process Before, During, and After the COVID-19 Pandemic

**DOI:** 10.7759/cureus.74952

**Published:** 2024-12-02

**Authors:** Uday Mahajan, Mahmoud Bhingraj, Sonu Mehta, Edward Spurrier

**Affiliations:** 1 Trauma and Orthopaedics, Queen Elizabeth Hospital Birmingham, Birmingham, GBR; 2 Trauma and Orthopaedics, University Hospitals Sussex NHS Foundation Trust, London, GBR; 3 Trauma and Orthopaedics, Glan Clwyd Hospital, Rhyl, GBR

**Keywords:** medical education, orthopaedic surgery, specialty training, st3 selection, trauma and orthopaedics

## Abstract

The selection process for Specialty Training Level 3 (ST3) in trauma and orthopaedics is a pivotal yet challenging step for aspiring orthopaedic surgeons. Drawing on personal experience and research, this paper aims to provide a comprehensive analysis of the ST3 selection process, outlining the key stages, including application submission, portfolio evaluation, and interview performance. With recent changes, such as evolving self-assessment scoring criteria and the introduction of a communication station in 2024, candidates face an increasingly dynamic and competitive landscape. This review study explores the challenges faced by both candidates and authorities, including adapting to sudden changes, maintaining fairness, and ensuring robust assessments. By offering insights into the evolution of the selection process and practical advice for preparation, this paper seeks to guide future candidates in navigating the complexities of this rigorous process and contribute to a greater understanding of what is required to succeed in this highly sought-after specialty.

## Introduction and background

The selection process for Specialty Training Level 3 (ST3) in trauma and orthopaedics serves as a crucial step for candidates aspiring to enter this competitive specialty [1]. Traditionally, the process involved in-person assessments, including portfolio reviews, clinical scenarios, and interviews, to evaluate candidates comprehensively [2,3]. However, the COVID-19 pandemic prompted a swift transition to remote assessments, altering the selection framework and introducing new challenges. This study explores the impact of these changes, focusing on the difficulties faced by candidates in adapting to remote assessments, the annual adjustments in self-assessment scoring, and the uncertainties surrounding interview cut-offs. Additionally, the 2024 introduction of a communication station underscores the evolving nature of the selection process. By reflecting on these adaptations, this research offers insights into how the ST3 trauma and orthopaedics selection process has evolved and provides guidance for candidates in preparing for the dynamic challenges of this demanding field.

## Review

This study employed a literature review to investigate the selection process for higher ST3 in trauma and orthopaedics in the UK. Online databases and search engines were used to retrieve relevant literature using keywords such as "ST3 trauma and orthopaedics", "specialty training in trauma and orthopaedics", "orthopaedic surgery training selection", and "higher speciality training trauma and orthopaedic". Inclusion criteria encompassed publications focused on the ST3 selection process in trauma and orthopaedics, written in English, from peer-reviewed journals, conference proceedings, and official reports published within the last five years. Articles were screened based on titles and abstracts for relevance, and selected publications were subjected to detailed analysis. Thematic analysis was applied to extract key insights, including candidate selection criteria, assessment methods, and potential challenges. Ethical considerations were observed, as the study relied solely on publicly available data. Limitations include potential publication bias and exclusion of unpublished or proprietary information.

This literature review explores encompassed sources related to the selection process for ST3 in trauma and orthopaedics [1-9]. The reviewed literature consisted of official websites, research articles, and YouTube videos. Among the sources were person specifications and entry criteria for ST3 trauma and orthopaedic surgery for the 2025 recruitment by Health Education England Yorkshire and the Humber, offering insights into required qualifications and attributes [4]. Additionally, YouTube videos featured interviews and advice from experts and previous candidates, covering various aspects of the application process and updated information for the 2023 intake [5,6]. Research articles published provided in-depth examinations of the national recruitment process and common characteristics of successful candidates during specific periods [1-3]. A resource from the British Orthopaedic Association (BOA) detailed the 2020-2021 selection process, including adaptations made due to the COVID-19 pandemic [7]. The literature reviewed presented a comprehensive overview of the selection process, incorporating perspectives from multiple sources and regions.

The candidate selection criteria and assessment process for ST3 in trauma and orthopaedics involved multiple stages, including application longlisting, shortlisting, and the interview process. The application process likely began with longlisting, where candidates submitted their applications, including relevant academic qualifications, surgical experiences, and other supporting documentation. Subsequently, shortlisting would have been conducted to select the most promising candidates based on predetermined criteria, such as academic achievements, research experience, and personal qualities. The interview stage comprised three stations, each designed to assess specific aspects of the candidate's suitability for the program. The first station, the portfolio station, likely evaluated candidates' ability to showcase their professional achievements, research contributions, and commitment to the specialty. The clinical station, on the other hand, would have assessed candidates' clinical skills, problem-solving abilities, and patient management capabilities through simulated patient scenarios or case-based discussions. Lastly, the prioritization station would have tested candidates' ability to make sound decisions and prioritize tasks, reflecting the challenges they might encounter in real-world clinical practice [8,9]. Overall, the selection process aimed to identify candidates with a well-rounded skill set, combining academic excellence, clinical expertise, research accomplishments, and the essential qualities needed to excel in trauma and orthopaedics. By evaluating candidates through a multi-station interview format and scrutinizing their portfolios, the process sought to ensure the selection of individuals who would thrive and contribute significantly to the specialty training program.

Over the past years, the selection process for ST3 orthopaedics has experienced significant variations, with distinct changes observed before, during, and after the COVID-19 pandemic. Before the pandemic, the number of applications remained relatively stable, accompanied by fluctuations in the chances of being appointed, suggesting competitive dynamics. The conventional face-to-face assessments, including multiple assessors for each station, aimed to comprehensively evaluate candidates' suitability for the specialty training program. However, the COVID-19 outbreak necessitated adaptations, leading to a shift towards remote assessments in 2020. The portfolio station emerged as the primary method for selection, with a consequent reduction in the number of appointments offered due to the pandemic's impact on healthcare services. This challenging period created difficulties for candidates in showcasing practical skills remotely and intensified competition, resulting in a lower chance of appointment. However, post pandemic, the selection process continued with remote assessments, and the chances of appointment improved compared to the pandemic year, showing a return to a more stabilized selection landscape.

The challenges and limitations faced by both candidates and authorities were pronounced during these dynamic periods. For candidates, the shift to remote assessments during the pandemic posed hurdles in adequately demonstrating clinical skills, potentially impacting their chances of selection. The reduced number of available slots for interviews heightened competition and contributed to heightened stress and anxiety among applicants. On the other hand, authorities encountered the task of rapidly adapting to the pandemic's uncertainties, implementing remote assessment methodologies while ensuring fairness and accuracy. Balancing the needs of candidates and healthcare institutions in the face of limited positions was a delicate responsibility. Post pandemic, the persisting challenges included conducting effective remote assessments, ensuring standardized evaluations, and addressing potential technological issues. The varying chances of appointment in different years necessitated consistent evaluation criteria and transparent processes. Despite these challenges, continuous efforts by authorities to refine the selection process and support candidates have contributed to the resilience of the ST3 orthopaedics specialty training program, demonstrating an adaptive approach towards identifying deserving and qualified candidates for this prestigious medical field.

The evolution of the ST3 selection process in trauma and orthopaedics over the past few years has introduced new dynamics and challenges for candidates, particularly in the wake of the COVID-19 pandemic. While the number of available positions may vary from year to year, it is important to clarify that the overall number of positions has not been significantly reduced. Instead, candidates have faced increased competition and uncertainty due to fluctuations in application numbers and appointment chances. Additionally, the self-assessment scoring system has seen slight adjustments annually, contributing to uncertainty around the cut-off scores required for securing an interview. This variability adds an extra layer of complexity for candidates as they prepare their applications, emphasizing the importance of staying informed about the latest changes in the selection process.

From a candidate's perspective, the introduction of new assessment components can also be challenging. Notably, the 2024 selection process saw the last-minute introduction of a communication station, where candidates were required to handle patient communication scenarios. This sudden change underscores the need for candidates to be adaptable and well-prepared for unexpected challenges during the selection process. Planning and preparation are crucial, as is dedication to staying up-to-date with potential changes to the interview format or scoring criteria.

Looking ahead, trauma and orthopaedics remain a highly competitive specialty, and candidates must be prepared to tackle any last-minute changes with resilience and adaptability. Thorough preparation, adaptability, and a deep commitment to mastering the interview process are essential for success in this demanding field. Candidates must be ready to navigate last-minute changes and unexpected scenarios, while authorities continue to refine the selection process to maintain its rigor and fairness.

Interviewers and authorities have expressed a strong preference for returning to an Objective Structured Clinical Examination (OSCE)/Multiple Mini Interview (MMI)-style format seen in in-person interviews, which allows for a more comprehensive evaluation of candidates' skills and abilities. The challenges faced with previous online interview platforms, such as Microsoft Teams, made it difficult to implement an OSCE-style format effectively. However, with the introduction of Qpercom (Galway, Ireland) for the 2024 ST3 orthopaedic interviews, candidates can expect a more structured and rigorous circuit of interview stations that closely resembles the traditional in-person format [4]. This shift is anticipated to better differentiate between candidates and ensure that the most qualified individuals are selected for the program.

## Conclusions

The ST3 selection process in trauma and orthopaedics has undergone significant transformations, particularly in response to the challenges posed by the COVID-19 pandemic. These changes have introduced new complexities for candidates, such as fluctuating appointment chances, evolving self-assessment scoring systems, and the introduction of new assessment components like the communication station. As the process continues to evolve, the importance of thorough preparation and adaptability cannot be overstated. Candidates must be ready to face unexpected challenges and demonstrate resilience in a highly competitive environment.

By leveraging Qpercom, the interviews aim to combine the accessibility and flexibility of a virtual platform with the rigour and comprehensiveness of traditional in-person assessment methods. This ensures a fair, robust, and standardized evaluation of candidates. This structured approach will better differentiate between candidates, ensuring that those with the highest potential are selected for the specialty. Ultimately, success in this demanding field will require candidates to remain informed, dedicated, and fully prepared to meet the evolving demands of the selection process.
